# Science Diplomacy in Latin America and the Caribbean: Current Landscape, Challenges, and Future Perspectives

**DOI:** 10.3389/frma.2021.670001

**Published:** 2021-06-17

**Authors:** Marga Gual Soler

**Affiliations:** ^1^SciDipGLOBAL, Palma, Spain; ^2^National Autonomous University of Mexico, Mexico City, Mexico

**Keywords:** science diplomacy, Latin America & Caribbean, South-South cooperation, science for peace, sustainable development goals, capacity building, foreign policy, global governance and multilateralism

## Abstract

Science, technology, and innovation are taking center stage in international affairs and increasingly influencing the geopolitical dynamics and a country's standing on the global stage. New scientific and technological advancements are acquiring greater strategic relevance to ensure competitive advantages in the twenty-first century global order. At the same time, international scientific collaboration contributes to generating and democratizing knowledge and improving relations between countries as a “soft power” tool to coordinate science-based solutions to transboundary problems, and to build bridges between countries with tense diplomatic relations. Science diplomacy is not a new concept, but most of its intellectual foundations and practical applications have emerged in the Global North. This article describes the diverse approaches, policies and practices adopted by Latin American and Caribbean countries at the national, sub-national, and regional levels. It analyzes their successes and challenges and identifies opportunities to guide the region toward a common science diplomacy strategy to achieve sustainable development through incorporating science as a permanent element in the foreign policy toolkit of Latin American nations. By documenting and illuminating best practices in the region, this article also seeks to balance the emphasis that has so far been largely concentrated on the regions of Europe and North America and contribute to future efforts and strategies for the development of sustainable science diplomacy mechanisms at the national, regional, North-South and South-South levels.

## Introduction

Science diplomacy is gaining relevance as an essential tool to tackle global challenges that have a scientific dimension, do not respect national borders, and no country can solve alone (Ruffini, 2017). To reverse climate change, achieve sustainable development, provide food and clean energy to billions of people, restore biodiversity, and prevent and tackle global health crises such as the COVID-19 pandemic, greater coordination between the spheres of science and foreign affairs will be key (S4D4C, 2019). By aligning scientific and diplomatic agendas, nations can attract scientific talent, strengthen national research, and innovation systems and competitiveness, provide avenues for greater participation of scientists in the formulation of public policies, coordinate integrated solutions to common problems, and tend bridges between countries with tense or non-existent diplomatic relations (Quevedo, 2013; Gluckman et al., 2017).

The countries of Latin America and the Caribbean have a wide range of bilateral, regional, and global scientific cooperation instruments to strengthen and complement national research capacities (OEI, 2012), but science still plays a secondary role in policy in general and foreign policy in particular (Gual Soler, 2020a). Despite numerous multilateral initiatives “on paper,” the region has failed to take full advantage of the opportunities and additional benefits that scientific collaboration offers to facilitate international relations, coordinate common actions in the face of transnational challenges, and achieve shared development objectives (Gual Soler, 2014). This article reviews the evolution and current landscape of science diplomacy, starting with a global outlook before focusing on Latin America and the Caribbean. The analyses and recommendations are informed by the author's own perspective after a decade of direct engagement as a policy advisor, researcher, lecturer, and trainer in science diplomacy across Latin American and Caribbean nations and institutions.

## What is Science Diplomacy?

Throughout history, science has served as a meeting point to build alliances between countries under political tensions (Müller and Bona, 2018) in support of diplomatic agreements in areas of global health, biodiversity conservation, ocean governance, water resource management, nuclear non-proliferation, energy security or climate change, among many others. According to Costa Rican scientist Marino Protti Quesada (2018), “*there are areas of the planet, such as the open seas and deep ocean floors, the outer atmosphere and extraterrestrial space, which are used by many countries for their exploitation and for scientific research. These are fertile grounds for international conflicts, but also for the peaceful coexistence of nations if there are international treaties that regulate their use and guarantee peaceful coexistence.”* Examples include the Antarctic Treaty of 1959 that dedicated Antarctica exclusively for peaceful scientific research, the Montreal Protocol of 1987 that achieved an unprecedented agreement between the scientific community, governments, and industry to eliminate the chemicals that damage the ozone layer, and the 2015 Paris Agreement that aligned 195 nations in a common goal to reverse climate change (Lewis et al., 2017; Paglia, 2021).

Despite this long tradition, the concept of science diplomacy and its applications began to gain traction well into the twenty-first century (Ruffini, 2020b). The term became popular after the Royal Society and the American Association for the Advancement of Science (AAAS) published in 2010 the first definition and theoretical framework for science diplomacy (The Royal Society & the American Association for the Advancement of Science, 2010). The report “*New Frontiers in Science Diplomacy”* organized science diplomacy in three main axes:

*Science in Diplomacy* refers to scientific support to foreign policy on bilateral and multilateral issues where science and technology are important, such as intergovernmental platforms on climate change and biodiversity, the management of shared natural resources and transboundary ecosystems, the coordinated management of global health treats, or the governance of global environmental commons such as Antarctica or the High Seas.

*Diplomacy for Science* refers to the diplomatic apparatus facilitating scientific collaboration between countries and promoting academic mobility and attraction of talent, knowledge, and innovations to improve the country's competitiveness. Diplomats pave the way for scientific cooperation at various levels, from the processing of visas and research permits for foreign students and researchers, to the negotiation of agreements and treaties for the construction and operation of large-scale scientific infrastructures, such as large telescopes or physics laboratories.

*Science for Diplomacy* refers to scientific cooperation as a soft power tool to improve international relations and establish communication channels between countries that are experiencing difficult relations in the political, economic, human rights, trade, or other spheres. In this case, international cooperation in science generates diplomatic benefits as well as advances in knowledge.

This initial conceptualization was largely predicated on the universality of science and its ability to cross borders and connect societies to address common challenges. This somewhat idealized vision has evolved over the years toward more pragmatic approaches, with the use of science, technology, and innovation at the bilateral and multilateral levels to meet both global goals and national interests (Ruffini, 2020b). In 2018, a group of scientific advisers to the foreign ministries of the United States, United Kingdom, Japan, and New Zealand proposed a new classification of science diplomacy actions based on the motivations of foreign ministries (Gluckman et al., 2017). These include improving a country's capacity for innovation and competitiveness through access to knowledge, markets, and technology abroad while attracting talent and investment; solving transboundary problems, such as the management of cross-border water resources; and addressing global challenges on a planetary scale, such as reducing carbon dioxide emissions or eliminating plastics in the ocean.

In the third decade of the twenty-first century, the strategic vision for science diplomacy is more and more oriented toward the commercial sphere, expanding its scope from the ministries of foreign affairs to cross-cutting strategies encompassing the ministries responsible for trade, education, environment, technology, health, or economy (Gluckman et al., 2017). Furthermore, science diplomacy is not limited to state actors or national governments: sub-national and supranational entities, the private sector, and civil society are becoming increasingly involved in its processes and activities (Melchor, 2020). Regardless of the approach, putting science diplomacy into practice requires new configurations and models of national, regional, and global collaboration between diplomatic institutions and the scientific community. To accomplish this, a myriad of actions are emerging that range from including scientific personnel in foreign ministries and embassies, exposing diplomats to science and technology issues and/or training scientists to communicate their science to decision makers, to the establishment of new institutions and professions dedicated to this matter.

## Who are the Science Diplomacy Actors?

Given the complexity and speed of scientific and technological developments, many governments have recognized the need to understand the diplomatic implications of scientific innovations and incorporate science, technology and innovation into their foreign policy structures (Turekian and Kishi, 2017). In the last decade, ministries of foreign affairs, diplomatic services, international organizations, and universities have begun to coalesce into an ecosystem to align international cooperation in science with foreign policy objectives. The European S4D4C project^1^ classifies the main players in the science diplomacy ecosystems as follows:

*Governments* are responsible for the design and implementation of national policy agendas and the coordination of scientific, environmental and health policies with foreign, development, defense, or trade policies. They involve ministries, embassies, research funding agencies as well as state and municipal governments. They establish bilateral and multilateral agreements for scientific cooperation with priority countries, articulate networks of scientists abroad, and appoint specialized functions to implement science diplomacy (Melchor, 2020). Two of the best characterized and institutionalized functions in science diplomacy are science attachés in embassies and diplomatic missions, and scientific advisers to foreign ministries, although there is considerable variation between countries in the background and recruitment mechanisms of these professionals. For example, science cooperation can be framed under the area of economic cooperation, or as part of cultural and academic affairs, depending on how a country's foreign service is organized. Some countries recruit academics who temporarily serve as scientific attachés, while others hire nationals of the country with local knowledge (Ittelson and Mauduit, 2019). The most common model of scientific advice in foreign ministries is the Chief Science Advisor, a direct advisor to the foreign minister who works alongside a multidisciplinary team of specialists in different fields of science and technology (Gluckman, 2016).

*International organizations* propose and raise transnational issues of a scientific nature and of global interest in the agendas of member states and generate science-policy interfaces to achieve multilateral solutions to common problems (Van Langenhove, 2016). They employ international civil servants, consultants, and advisors working at the science-diplomacy nexus interfaces. Examples include the United Nations System and other multilateral and supranational organizations such as the Organization for Economic Cooperation and Development, the African Union, the Inter-American Institute for Global Change Research, or the European Commission.

*Academic sector*: Universities, research centers, scientific-technical infrastructures, national academies of sciences, and scientific societies contribute knowledge toward solutions to national and global challenges and create spaces for dialogue and collaboration between scientists from different countries (Lyons et al., 2021). In some countries, academic experts contribute to diplomatic negotiations and are seconded to foreign ministries, development agencies, and embassies to serve as science counselors or attachés for a period of time and then return to their university positions^2^.

*Private sector*: Companies seeking to access knowledge, technology, and innovation abroad often serve the agendas and interests of their home countries and promote services and products aligned with the country strategy and brand. Innovation diplomacy strategies are increasingly supporting the internationalization of startups and governments are setting up diplomatic representations to tech hubs in the face of the growing geopolitical importance of transnational technology companies and their critical role in the global governance of frontier technologies (Gual Soler, 2020a).

*Civil society:* A growing number of are NGOs, international networks, scientific associations, and private foundations specialize in building bridges between science, politics, and society. They elevate topics for national and international agendas and support science diplomacy through research, cooperation, training, and advisory programs and projects. For example, the AAAS Center for Science Diplomacy played a key role in establishing science diplomacy training programs around the world and promoting bilateral cooperation between scientists from the United States and Cuba, Iran, and North Korea amid long standing political tensions^3^. The International Network for Government Science Advice (INGSA) brings together science advisers to foreign policy to exchange experiences, successful models, and promote cooperation and training^4^.

## Science Diplomacy Strategies Around the World

Science diplomacy is implemented through diverse instruments, including bilateral and multilateral research agreements, collaboration networks, representations abroad, training programs, and in general any activity that involves science and foreign policy actors (Flink and Schreiterer, 2010). In several countries, a strong commitment to science diplomacy led to the redesign of certain structures of their Ministries of Foreign Affairs.

For example Denmark was a pioneer of *techplomacy* with the appointment of the first ambassador to Silicon Valley to promote digital diplomacy with high-tech companies as a priority for foreign policy, as well as raising awareness of the risks of disruptive technologies and digital divides^5^. This model gained traction quickly among other nations such as France or Germany. In 2016 Spain launched its Strategy for Science, Technology and Innovation Diplomacy jointly promoted from the Ministry of Science and Innovation and the Ministry of Foreign Affairs (Gobierno de España, 2016). Its objectives range from organizing the scientific diaspora, promoting Spanish scientific and technological advances abroad, and introducing scientific training in the diplomatic academy. Scientific coordinators and science diplomacy interns were installed in key embassies such as London, Berlin, and Washington. France created a Department of Global Affairs in the Ministry of Foreign Affairs that includes an office for scientific mobility and attraction policies, with a network of hundreds of science attachés and volunteers deployed in the French representations abroad to link them with French research institutes, companies and centers of competitiveness^6^. Similarly, Japan deployed specialized science and technology officials to more than 20 representations abroad with the aim of expanding access to resources for research outside its borders. The United Kingdom approaches science diplomacy for international economic positioning and increasing its soft power in new countries through a Science and Innovation Network of 90 officers in 28 countries and 47 cities through its network of embassies and consulates^7^. Switzerland articulates the Swissnex network to link innovation hubs with the science and technology offices of the Swiss embassies to strengthen Switzerland's profile as a partner^8^ has recently appointed a Special Envoy for Science Diplomacy^9^. A similar posting was created in the Czech Republic^10^. The Netherlands launched a specific fund for scientific cooperation with countries with which it seeks to strengthen its diplomatic relations^11^.

Beyond this mosaic of national approaches, supranational and subnational strategies are on the rise. The European Union gave a high profile to science diplomacy in 2015 (Moedas, 2016) and in 2020, the European External Action Service (EEAS), the foreign policy arm of the European Union, appointed its first science advisor. At the state, region, and province levels, there are science and innovation diplomacy strategies in São Paulo in Brazil^12^, Quebec in Canada^13^, and Wallonia in Belgium, and at the city level in Geneva, Barcelona, Boston, New York, Shanghai, and Mexico City (Roig, 2019). Some of these cities have created 'science and technology diplomacy circles' that bring together those responsible for science, technology, and innovation from diplomatic missions and international organizations both in capitals and in innovation hubs^14^. This is turning cities into geopolitical actors, serving as laboratories of innovation through scientific, technological, and cultural exchange and driven by innovative public-private partnerships.

## The Science Diplomacy Landscape in Latin America and the Caribbean

The debate on science diplomacy and its applications has been developed mainly in English, dominated by North America, Europe, Japan, or New Zealand (Turchetti et al., 2020) and therefore the resources in Spanish on this concept are still limited. The countries of Latin America and the Caribbean have a wide range of bilateral, regional, and global scientific cooperation instruments to strengthen and complement national research capacities (OEI, 2012). However, despite numerous multilateral initiatives, the region has not been able to take full advantage of the opportunities and additional benefits that scientific collaboration offers to facilitate international relations, coordinate common actions in the face of transnational challenges, and achieve collective sustainable development goals. Political instability, ideological fragmentation, budget problems, and the multiplicity and redundancy of high-level forums with different memberships and configurations, each with its own science and technology commission^15^, have limited the effectiveness and relevance of multilateral scientific initiatives.

But in 2015, the UNESCO Regional Office of Science for Latin America and the Caribbean introduced science diplomacy in its agenda for sustainable development^16^. Since then, science diplomacy has risen on the agendas of several Latin American countries and multilateral organizations, and has been promoted in high-level regional training and dialogues. Argentina, Brazil, Chile, Costa Rica, Cuba, Mexico, and Panama are some of the countries that have started, reinforced and labeled their activities the science-foreign policy interface as “science diplomacy” actions, adopting diverse strategies to incorporate science, technology and innovation into their foreign policy structures (Gual Soler, 2020b). These were highlighted recently at the 2021 edition of the Latin American Open Science Forum (Foro CILAC) promoted by UNESCO (de Ambrosio, 2020).

### National Approaches

Panama, Colombia, and Costa Rica are taking steps to institutionalize their science diplomacy strategies within the science and foreign ministries. In 2018 Panama became the first Latin American nation to define a national strategy. The “Strategy of scientific, technological and innovation diplomacy as an instrument of 21st century diplomacy,”^17^ jointly promoted by the Ministry of Foreign Affairs and the National Secretariat for Science, Technology, and Innovation, sought to leverage Panama's strategic location as a connecting hub in the Americas, its world-class tropical biodiversity and expertise in tropical medicine, and set out to equip Panamanian diplomats with knowledge in science, technology, and innovation to align Panama's foreign policy with the UN Sustainable Development Goals. In 2021, science diplomacy will be incorporated in the new Science Law, paving the way for the strengthening of scientific advice at both the domestic and international levels (Gittens et al., 2021).

With the creation of the new Ministry of Science, Technology, and Innovation in 2020, Colombia is in the process of creating a national science diplomacy strategy, which proposes the creation of 9 nodes in strategic countries (including border areas with Brazil, Panama, and Peru), a greater articulation between the scientific diaspora and the Colombian scientific and academic communities with international networks, and capacity building in science diplomacy, both within the government and in other entities and actors^18^. The positioning of Colombia in South-South cooperation scenarios is also part of the strategy, taking into account its new role in the region and its potential to support countries with fewer capacities after its entry into the OECD in April 2020^19^.

Costa Rica has long been a leader in climate negotiations from the 1992 Earth Summit to the 2015 Paris Agreement, whose architect was the Costa Rican diplomat Christiana Figueres. In 2014 Costa Rica appointed for the first time a scientist as ambassador to the United States, who launched an ambitious bilateral scientific cooperation agenda in the areas of water, public health, disaster prevention, and remote sensing. Costa Rica also promotes the peaceful application of technology related to disarmament and international security, including the peaceful uses of nuclear energy, security in space and cyberspace, and the use of new technologies such as artificial intelligence at the service of peace and sustainable development^20^. In 2019 the Ministry of Foreign Affairs, through its Manuel María de Peralta Foreign Service Institute and the National Academy of Sciences of Costa Rica (ANC), began institutional efforts to link diplomats with researchers, and in 2020 initiated the formal structuring of a science diplomacy strategy under the Economic Diplomacy Process (López-Vergès et al., 2021).

However, not all efforts that fall under the science diplomacy umbrella are labeled as such, which complicates their analysis and categorization (da Silva et al., 2021). For example, Brazil and Chile have well-established science cooperation departments within the foreign ministry that only recently started using the terminology. In Brazil, the Department of Science and Technology in the Ministry of Foreign Affairs has an Innovation Diplomacy Program implemented both at the federal and subnational level from the state of São Paulo^21^. Chile has a Directorate of Energy, Science and Technology and Innovation in the Ministry of Foreign Affairs which seeks to link Chile's policies in energy, science, technology, and innovation with foreign policy through strategic alliances with key countries, international organizations and other relevant actors for strengthening and complementing national capacities in these areas^22^. It's worth noting that only Brazil and Chile have formalized the figure of science attaché in their embassies, and to date no Latin American country has fully institutionalized the figure of scientific advisor to the Ministry of Foreign Affairs, although there are variations such as the appointment of an Ambassador for Science, Technology, and Innovation in Bolivia^23^ and the opening of Uruguay's first technology consulate in San Francisco, USA^24^.

In the “science for diplomacy” dimension, Argentina and Cuba are good examples of using science to build diplomatic relations or to ease tensions in other areas. Argentina has managed to forge fruitful scientific cooperation with the United Kingdom despite the complicated diplomatic relations of the last decades over the Malvinas/Falkland Islands in areas like agri-technology, advanced materials and nanotechnology, ICT, life sciences, marine science, and palaeontology (Grimes, 2018). International scientific cooperation has also contributed to the consolidation of long standing interactions that can overcome the gaps associated with changes in the national government. For example, it is a usual practice by Argentine ambassadors to start with a scientific mission after they arrive at a new destination because it is usually an area devoid of potential conflicts (Barañao, 2016). Cuba is one of the countries with the longest tradition in scientific and medical diplomacy in the region, based on its advanced biotechnology industry and a robust health system^25^. Since 1963 the country has sent more than 400,000 medical professionals in 164 missions to countries in Africa, America, the Middle East, and Asia, in addition to providing humanitarian aid in cases of catastrophes, emergencies, and epidemics such as Ebola and more recently COVID- 19. This has allowed Cuba to gain international prestige and political capital, reflected for example in the votes against the United States embargo in the UN General Assembly (Malacalza, 2016). The Cuban Academy of Sciences has been instrumental in facilitating scientific cooperation between Cuba and the United States on issues of common interest such as hurricanes and infectious diseases, due to the lack of official diplomatic channels during various periods of relations between the two countries. The sustained cooperation between the Cuban Academy of Sciences and non-governmental scientific institutions such as AAAS and the US National Academy of Sciences paved the way for the reopening of diplomatic relations in 2015 (Pastrana et al., 2018).

A growing trend is the orientation of science diplomacy toward strategic sovereignty in the space, energy, nuclear or maritime spheres. For example, the Pampa Azul initiative in Argentina articulates the work of seven ministries, including science and foreign relations focused on the Argentinian Sea^26^. Recently, Argentina and Mexico agreed to lead the creation of a Latin America Space Agency^27^. Thanks to its privileged geographical position for astronomical observation and access to Antarctica, Chile bases a significant part of its strategy on attracting large international research projects in “natural laboratories” in the Andes and the Chilean Antarctic bases. And a recent proposal aims to create the ANDES Lab, an underground binational physics laboratory between Chile and Argentina with a model similar to the European CERN^28^.

In recent years, Ecuador, Peru, and Uruguay have also begun promoting international research collaborations and mobility to enhance their visibility in the global stage and access economic, human and material resources not available domestically (Belli and Baltà, 2019).

Other initiatives in progress in the region include the launch of a scientific, technological, and business innovation diplomacy program in the Dominican Republic^29^.

### Regional Approaches

At the regional and bi-regional levels, two instruments of note are the Ibero-American Program for Science and Technology for Development (CYTED) and the Inter-American Institute for Global Change Research (IAI). Both programs, born as North-South scientific collaboration networks promoted by Northern countries, Spain, and the United States, evolved into more horizontal South-South cooperation schemes and have contributed to the regional integration of Latin America, creating spaces for the incorporation of science in public policy, decision-making, and governance, resulting in greater regional cohesion and harmonization of science policy mechanisms (Gual Soler, 2014).

The Latin America Open Science Forum (Foro CILAC) promoted by UNESCO is a bi-annual event rotating different cities across the region for dialogue and regional cooperation to devise a common horizon in science, technology, and innovation for Latin American countries to achieve the 2030 Agenda^30^. Science diplomacy has been a central theme in both high-level sessions and training workshops since the launch of CILAC in 2016, and a policy brief dedicated to science diplomacy in Latin America and the Caribbean was launched at Foro CILAC 2021 (Gual Soler, 2020b).

### Education and Training in Science Diplomacy

Most of the national and regional science diplomacy strategies described in this article include a training component to foster the new types of professionals capable of navigating the science-diplomacy nexus. The Diplomatic Academy of Chile Andrés Bello launched in 2019 a science diplomacy training track to promote the insertion of Chile in international research and innovation networks^31^. Argentina, Panama, and Mexico have also recently incorporated science, technology, and innovation modules into their diplomatic training (Gual Soler, 2020b). An important element of the Panamanian strategy was to incentivize the recruitment of STEM professionals to diplomatic careers, updating the rules and requirements for entry into the foreign service to accept graduates from any background^32^.

In the last decade, several international initiatives have emerged to formalize educational and curricular structures for the training of specialists in this interface. For example, The World Academy of Sciences (TWAS), in collaboration with AAAS, has offered since 2014 a landmark course in science diplomacy at its headquarters in Trieste, Italy, which has trained over 300 young scientists and diplomats from the Global South, Although fewer trainees from Latin America have attended these trainings, in comparison to other regions^33^, they have had an outsized impact in advancing science diplomacy in their countries (Gittens and Lopez-Verges, 2018; Gittens et al., 2021). Other organizations offering science diplomacy training include INGSA, the IAI, the United Nations Institute for Training and Research (UNITAR), and the Horizon-2020 funded EU Science Diplomacy Cluster^34^.

Training models and formats range from short intensive courses and workshops to exchange programs of several days or weeks between scientists and diplomats, typically including role-play diplomatic simulations and science-intensive negotiation exercises, case studies, and networking activities^35^. But beyond *ad-hoc* training and workshops, to train professionals in science diplomacy and prepare both communities to work with one another, the most effective approaches are experiential learning programs and mainstreaming science diplomacy in university curricula (Mauduit and Gual Soler, 2020).

For example, countries in North America and Europe offer scholarships, internships, and pairing schemes that provide immersive experiences for scientists in governments, embassies, and international organizations during their graduate or postdoctoral work to gain experience in a government or parliamentary office (Gual Soler et al., 2017). The IAI has recently launched the first program of this kind, a pilot Science, Technology, and Public Policy Fellowship (SteP) with fellows from Argentina, Mexico, Canada, and the US^36^, Fellows spend a year immersed in a policy setting to facilitate the incorporation of scientific knowledge into policy processes relevant to global environmental change and obtain professional development in science diplomacy, science advice, and science communication to learn to integrate diverse knowledge and experience in different sectors and countries in response to the critical challenges of global change in the Americas.

An important development in recent years has been the introduction of science diplomacy curricula at Latin American universities. In 2019, the University of São Paulo in Brazil established the São Paulo School on Science and Innovation Diplomacy^37^. In 2020, the Universidad Externado de Colombia included a science diplomacy module in the “New Diplomacies” course, and the National Autonomous University of Mexico (UNAM) launched a science diplomacy course in the International Relations undergraduate program. Argentina has invested in international research and training programs for Latin American scientists to strengthen regional integration by creating environments in which young scientists can establish personal bonds that can lead to future scientific cooperation at a regional level, while promoting a sense of social responsibility that is not usually emphasized in the scientific centers of excellence in the Global North (Barañao, 2016). Similarly, Cuba contributes to training human resources in Latin America through international and regional schools in various disciplines from public health to climate resilience^38^.

## Challenges and Barriers

As science, technology, and innovation take on a growing value in world diplomacy, it will be necessary to create more spaces for collaboration between both worlds. A key challenge is that most of the intellectual foundations, practical applications, and case studies of science diplomacy have emerged from the Global North. Although there are successful and replicable models, each country must build its own structures adapted to the government system and the (geo)political, economic, social and scientific context. In addition, science diplomacy remains a fluid concept, understood differently by different stakeholders, and can be explicit or implicit^39^—that is, many activities, policies, programs, and instruments can be considered science diplomacy actions without the label, making them difficult to systematize, institutionalize, and operationalize (Turchetti and Lalli, 2020).

Latin America is one of the most unequal regions in the world (CEPAL, 2016)^40^. where societies face different moments in their development stage, some with very low investment in research, with the exception of Brazil^41^. Despite many Latin American countries having sophisticated STI policy instruments in place and strong growth in higher education, human resources development, and scientific production in recent years (UNESCO Science Report: Towards 2030, 2015), an absence of a tradition of evidence-based decision-making is still pervasive. It is common for the foreign ministry and the science ministry (if the country has one) to rarely interact, complicating the alignment between science policy and foreign policy. From a governance perspective, most Latin American diplomatic institutions were founded in the nineteenth century, while the institutionalization of science began in the second half of the twentieth century. But on the other hand, the region harbors a growing policy interest in indigenous knowledge and is stepping up investment in sustainability-related sciences. For example, the share of scientific articles focusing on indigenous knowledge has grown in all Latin American countries and is much higher in countries like Bolivia, Colombia, Guatemala, and Nicaragua than in developed countries (UNESCO Science Report: Towards 2030, 2015).

Another challenge is that it is common to appoint political appointees in diplomatic posts, often from the business sector (Acosta, 2006), which further complicates access from non-traditional backgrounds (e.g., STEM) to careers in foreign affairs. Connecting science and diplomacy requires a reconfiguration of the learning and professional development pathways of both communities and the participation of professionals who perform a variety of functions that often do not fit with traditional careers in science or international relations, as the necessary knowledge, skills, and capacities of its professionals are not yet fully defined and are highly context-dependent (Mauduit and Gual Soler, 2020). Furthermore, most countries outside the Global North lack non-governmental institutions that can act as a bridge between government and academia. These are usually best placed to advance science diplomacy and act as neutral intermediaries, especially between countries that do not have official diplomatic relations (Bednarek et al., 2018).

In countries with institutionalized science diplomacy such as the United States or the United Kingdom, many professionals with scientific training, most of them with PhDs, occupy full-time positions in the foreign ministry or embassies (Gual Soler et al., 2017). This model is problematic in the Latin American context, where many countries have an insufficient number of researchers (only 3.6% of global researchers)^42^, so those who stand out are constantly invited to form part of expert committees from various areas of government (Gittens and Lopez-Verges, 2018), complicating both their daily practice as researchers, as well as their understanding of the complexity of policy and diplomacy processes. Many Latin American countries also face have legal or bureaucratic barriers to entering the diplomatic career from a science, technology, or engineering background, in addition to cultural barriers, lack of awareness among graduates about the range of professional options available, and resistance from academia to the idea that a scientist can work in other sectors.

## Seven Steps to Strengthen Science Diplomacy in Latin America and the Caribbean

Globalization is reconfiguring traditional geographic and geopolitical boundaries. The transnational nature of the most complex problems requires international dialogues between multiple actors from different countries and regions at all levels of government—local, state, national, and supranational. Although the Latin American region suffers from deep inequalities, its countries, unlike other more heterogeneous regions such as Africa or Asia, share cultural, linguistic, historical, and religious traditions, which is an advantage for regional integration in the face of shared challenges such as endemic diseases or vulnerability to climate change (Barañao, 2016). The examples shown here indicate that science diplomacy is experiencing a surge in popularity in the Latin American region, but the greatest challenge will be to build sustained bridges between actors, policies, and functions that can survive political cycles. Seven steps can help the region advance toward stronger regional coordination structures in science diplomacy.

First, science diplomacy should be introduced as an interdisciplinary field of study and research in Latin American universities in both science and international relations programs, as well as including it as a fundamental pillar for the external projection of academic institutions^43^. All science and technology students should receive communication, negotiation and leadership tools, interpersonal and intercultural skills, and knowledge of global policy issues. Establishing the figure of *Diplomat in Residence* in universities, common in US higher education, can provide guidance and advice on science diplomacy careers to the academic community^44^.

Second, governments should create scholarship programs, internships, and exchange schemes between researchers, public officials and diplomats, and articulate networks of scientists abroad to strengthen national scientific systems and promote “brain circulation.” They should also establish regular interministerial commissions for science diplomacy to foster direct communication between foreign ministries, ministries of science, trade, environment, energy and other actors, including non-governmental entities, to align science policy with foreign policy in a whole-of-government approach^45^.

Third, foreign services need structural, institutional, and educational changes, including promoting access to the diplomatic career and public service of professionals with training in science, technology, engineering and mathematics, introduce modules on science and technology in diplomatic training, establish permanent scientific advisory structures in ministries of foreign affairs, and creating the figures of science attaché in diplomatic missions and international organizations. These can include not only national governments, but sub-national entities such as city and regional governments.

Fourth, regional organizations must review and find synergies between spaces and commissions dedicated to scientific cooperation in the different regional and subregional forums to avoid multiplicities and redundancies.

Fifth, strengthening the role of the private sector, until now largely absent in science diplomacy spaces, as businesses are key drivers of the necessary transformations toward the Sustainable Development Goals (e.g., technology for education and communication, artificial intelligence, clean energy).

Sixth, new hybrid institutions should be created outside governments and academia at the national and regional level tasked with raising awareness, networking and training of different actors on science diplomacy issues, including close collaboration with science journalists and the media to contextualize science diplomacy to the Latin American reality and transmit its value to society.

Finally, all these efforts should converge in a regional network that articulates ministries, diplomatic academies, research agencies, universities, academies of science and other relevant entities to set a common agenda, exchange experiences, strengthen capacities, and coordinate actions, as well as connect with other international science diplomacy networks.

Cultivating better relations between the scientific and foreign policy arenas is an imperative for Latin America and Caribbean nations and institutions to ensure that science, technology, and innovation are engines of sustainable development and the region is more resilient to future crises^46^. Only by breaking the silos between governments, universities, the private sector and civil society will science, health, and the environment become true global public goods to achieve the 2030 Agenda.

## Author Contributions

The author confirms being the sole contributor of this work and has approved it for publication.

## Conflict of Interest

The author declares that the research was conducted in the absence of any commercial or financial relationships that could be construed as a potential conflict of interest.

## Footnotes

^1^www.s4d4c.eu

^2^https://sites.nationalacademies.org/PGA/Jefferson/index.htm.

^3^https://www.aaas.org/program/center-science-diplomacy/engagemen

^4^https://www.ingsa.org/divisions/spider/

^5^https://techamb.um.dk/en/techplomacy

^6^https://www.diplomatie.gouv.fr/en/french-foreign-policy/scientific-diplomacy/

^7^https://www.gov.uk/world/organisations/uk-science-and-innovation-network

^8^https://swissnex.org/

^9^https://www.eda.admin.ch/eda/en/fdfa/fdfa/aktuell/newsuebersicht/2021/02/science-diplomacy.html

^10^https://www.mzv.cz/jnp/en/foreign_relations/science_and_technology/index.html

^11^https://www.nwo.nl/en/news-and-events/news/2020/05/first-activities-awarded-in-science-diplomacy-fund.html

^12^https://2019.innscidsp.com/sao-paulo-framework-of-innovation-diplomacy (2019)

^13^http://www.scientifique-en-chef.gouv.qc.ca/en/dossiers/diplomatie-scientifique/stages-scientifiques-dans-les-representations-du-quebec-a-letranger/

^14^http://stdc-boston.com/

^15^Such as the Organization of American States (OAS), the Community of Latin American and Caribbean States (CELAC), the Union of South American Nations (Unasur), the Organization of Ibero-American States for Education, Science and Culture (OEI), Mercosur or the Central American Integration System (SICA).

^16^http://www.unesco.org/new/es/media-services/single-view/news/science_and_diplomacy_at_the_center_of_debate_for_sustainabl/

^17^https://mire.gob.pa/images/PDF/Lineamientos%20de%20la%20Estrategia%20de%20Diplomacia%20Cientifica%20-%20Rev%2031%20de%20mayo%20de%202019.pdf

^18^Equipo de internacionalización del Ministerio de Ciencia, Tecnología e Innovación de Colombia (Personal communication).

^19^https://www.oecd.org/newsroom/global-oecd-welcomes-colombia-as-its-37th-member.htm

^20^Ministerio de Relaciones Exteriores y Culto de Costa Rica. Personal communication.

^21^http://antigo.mctic.gov.br/mctic/opencms/institucional/Cooperacao_Internacional/Diplomacia-e-Inovacao-Cientifica-e-Tecnologica.html?searchRef=nanosseguran%C3%A7a&tipoBusca=expressaoExata

^22^https://minrel.gob.cl/politica-exterior/secretaria-general-de-politica-exterior/decyti

^23^https://comunicacion.gob.bo/?q=20200213/28796

^24^https://www.uruguayxxi.gub.uy/en/news/article/uruguay-inaugura-en-san-francisco-su-primer-consulado-tecnologico/

^25^https://www.coha.org/cuban-medical-diplomacy-when-the-left-has-got-it-right/

^26^https://www.pagina12.com.ar/276817-el-relanzamiento-de-pampa-azul-una-iniciativa-que-articula-e

^27^https://elpais.com/mexico/2020-11-21/alce-el-sueno-de-la-conquista-latinoamericana-del-espacio.html

^28^http://andeslab.org

^29^https://aduanasdigital.gob.do/2020/02/04/cancilleria-lanza-diplomacia-cientifica-tecnologica-e-innovacion-empresarial/

^30^http://www.forocilac.org

^31^http://www.academiadiplomatica.cl/index.php/academia-diplomatica-de-chile-potencia-la-diplomacia-cientifica/

^32^Decreto Ley 60 (2015). Decreto Ley 60, Pub. L. No. Ley 60, Rollo 620, Posición 698, artículo 3, Derecho Administrativo, Que Modifica y Adiciona Artículos a la Ley 28 de 1999, Sobre la Carrera Diplomática y Consular; Panamá

^33^https://twas.org/article/mainstreaming-science-diplomacy-global-south

^34^www.science-diplomacy.eu

^35^https://www.aaas.org/news/future-science-diplomats-receive-range-training-aaas

^36^http://www.iai.int/es/step

^37^https://2020.innscidsp.com/

^38^https://www.cubahora.cu/ciencia-y-tecnologia/ciencia-cubana-desarrollo-y-cooperacion

^39^This article only considers explicit science diplomacy actions, as defined by Van Langenhove (2016) in https://www.ies.be/files/Tools%20for%20an%20EU%20Science%20Diplomacy_by%20LukVanLangenhove.pdf

^40^https://www.cepal.org/es/comunicados/cepal-pese-avances-recientes-america-latina-sigue-siendo-la-region-mas-desigual-mundo

^41^Other than Brazil, no country in Latin America spends more than 1% of GDP on research and development https://en.unesco.org/sites/default/files/usr_7-7_gerd_lac_en.pdf

^42^https://en.unesco.org/sites/default/files/latin_americas_place_in_the_world_usr15.pdf

^43^https://www.fecyt.es/es/noticia/recomendaciones-para-la-diplomacia-cientifica-en-america-latina-y-el-caribe

^44^https://careers.state.gov/connect/dir/

^45^https://gesda.global/wp-content/uploads/2020/11/GESDA-SAB-9_Future-of-Science-Diplomacy.pdf

^46^https://www.scidev.net/america-latina/news/uruguay-y-covid-19-la-ciencia-al-servicio-de-la-poltica/
